# Role of ubiquitination in the occurrence and development of osteoporosis (Review)

**DOI:** 10.3892/ijmm.2024.5392

**Published:** 2024-06-26

**Authors:** Xiaoxia Fan, Rong Zhang, Guocai Xu, Peiyun Fan, Wei Luo, Chunmei Cai, Ri-Li Ge

**Affiliations:** 1 Research Center for High Altitude Medicine, Qinghai University, Xining, Qinghai 810000, P.R. China; 2 Key Laboratory of The Ministry of High Altitude Medicine, Qinghai University, Xining, Qinghai 810000, P.R. China; 3 Key Laboratory of Applied Fundamentals of High Altitude Medicine, (Qinghai-Utah Joint Key Laboratory of Plateau Medicine), Qinghai University, Xining, Qinghai 810000, P.R. China; 4 Laboratory for High Altitude Medicine of Qinghai Province, Qinghai University, Xining, Qinghai 810000, P.R. China; 5 Qinghai Provincial People's Hospital, Department of Endocrinology, Xining, Qinghai 810000, P.R. China

**Keywords:** ubiquitination, osteoporosis, bone marrow mesenchymal cell, osteoblast, osteoclast

## Abstract

The ubiquitin (Ub)-proteasome system (UPS) plays a pivotal role in maintaining protein homeostasis and function to modulate various cellular processes including skeletal cell differentiation and bone homeostasis. The Ub ligase E3 promotes the transfer of Ub to the target protein, especially transcription factors, to regulate the proliferation, differentiation and survival of bone cells, as well as bone formation. In turn, the deubiquitinating enzyme removes Ub from modified substrate proteins to orchestrate bone remodeling. As a result of abnormal regulation of ubiquitination, bone cell differentiation exhibits disorder and then bone homeostasis is affected, consequently leading to osteoporosis. The present review discussed the role and mechanism of UPS in bone remodeling. However, the specific mechanism of UPS in the process of bone remodeling is still not fully understood and further research is required. The study of the mechanism of action of UPS can provide new ideas and methods for the prevention and treatment of osteoporosis. In addition, the most commonly used osteoporosis drugs that target ubiquitination processes in the clinic are discussed in the current review.

## 1. Introduction

There are >8.9 million osteoporotic fractures occurring annually in the world (1). A total of >33% of women and 20% of men >50 years old will suffer an osteoporotic fracture at least once in their lifetime (2). With the increase in population aging, osteoporosis has become a chronic disease involving fractures, high disability and mortality and increased social and economic burden (3). Osteoporosis is a multifactorial disease characterized by bone loss and damage to the microstructure of the trabecular bone, leading to an increased risk of fracture and bone fragility (4,5). Therefore, identifying the pathogenesis of osteoporosis is of high importance.

Osteoporosis is closely linked to imbalances in bone homeostasis. The maintenance of bone homeostasis mainly depends on the balance between osteoclasts and osteoblasts; osteoclasts mediate bone resorption and osteoblasts mediate bone formation. In organisms, bone tissue constantly undergoes remodeling process through the synergistic action of bone marrow mesenchymal cells (BMSCs), osteoblasts, osteocytes and osteoclasts (6). BMSCs can multi-directionally differentiate into osteogenesis, adipogenesis and cartilage. BMSCs also promote calcium deposition and bone formation (7). Osteoblasts are derived from BMSCs and play a key role in the process of bone formation. They are mainly distributed on the surface of bone and synthesize bone matrix by secreting collagen and bone matrix proteins. Osteoblasts can synthesize and secrete the main components of bone, including collagen fibers, chondroitin sulfate, phosphoric acid and calcium. These secretions are important components of the bone matrix and play an important role in maintaining the structure and function of bone. Osteoclasts, originating from the hematopoietic monocyte-macrophage system, are a special type of terminally differentiated cells. They are involved in the process of bone remodeling through the uptake of bone matrix and minerals, as well as the secretion of organic acids and protein hydrolyzing enzymes, thus maintaining the normal structure and function of the skeleton (8). As osteoblast-derived cells, osteocytes maintain mature bone metabolism by mediating cytokine sensing, transduction and secretion to modulate bone resorption and formation (9). Under pathological conditions, osteoclast activity increases, while osteogenesis is either weakened or impaired, consequently destroying bone homeostasis. Over time, osteoporosis develops further.

During bone homeostasis and repair, Wnt/β-catenin, Hedgehog, bone morphogenetic protein (BMP-2)/Smad and phosphatidylinositol-3-kinase/protein kinase B and others are the major regulatory signaling pathways during skeletal growth and development. The Wnt/β-catenin signaling pathway plays a key regulatory role in bone development and formation by affecting the proliferation and differentiation of osteoblasts and osteoclast generation. Wnt is composed of 19 secreted glycoproteins and has the function of regulating cell growth, differentiation and apoptosis (10,11). The receptor activator of nuclear factor-κB ligand (RANK-L)/RANK/MAPK and the NF-κB axes are the major regulatory signaling pathways in osteoclast formation and function. RANKL integrates with the osteoclast surface receptor RANK, which in turn recruits tumor necrosis factor receptor-associated factor (TRAF) 6 and further activates multiple downstream signaling pathways, such as three mitogen-activated protein kinases (MAPKs) including p38 kinase (p38MAPK), extracellular signal-regulated kinase (ERK) and C-Jun N-terminal kinase (12,13).

**Figure 1:**
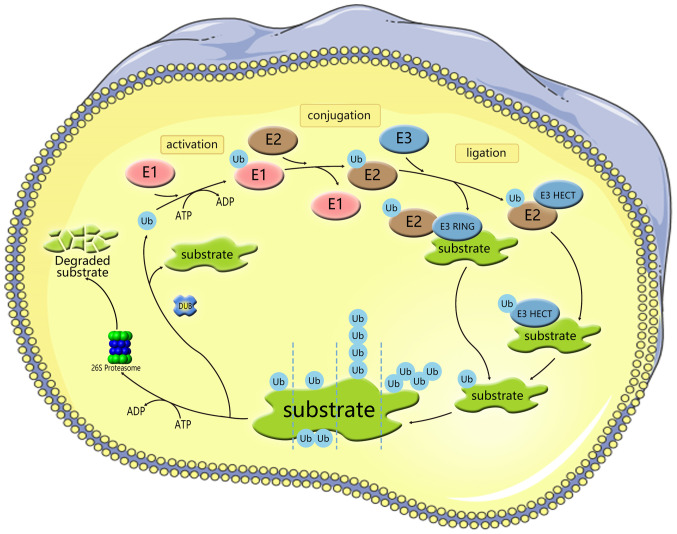
Protein ubiquitylation and DUBs. Ub is a small, highly conserved polypeptide composed of 76 amino acids. In the presence of ATP, Ub is activated and E1 begins to ubiquitinate. The E1 enzyme delivers the Ub protein to E2. Ub ligase E3 attaches the Ub bound to E2 to the target protein. Proteins can be ubiquitinated by several types, including mono-ubiquitination and poly-ubiquitination and are degraded in lysosomes or proteasomes, respectively. ^①^Mono-ubiquitylation; ^②^multi-ubiquitylation; ^③^poly-ubiquitylation type I; and ^④^poly-ubiquitylation type II. DUBs, deubiquitinases; Ub, ubiquitin; HECT, Homology to E6AP C-Terminus.

**Figure 2:**
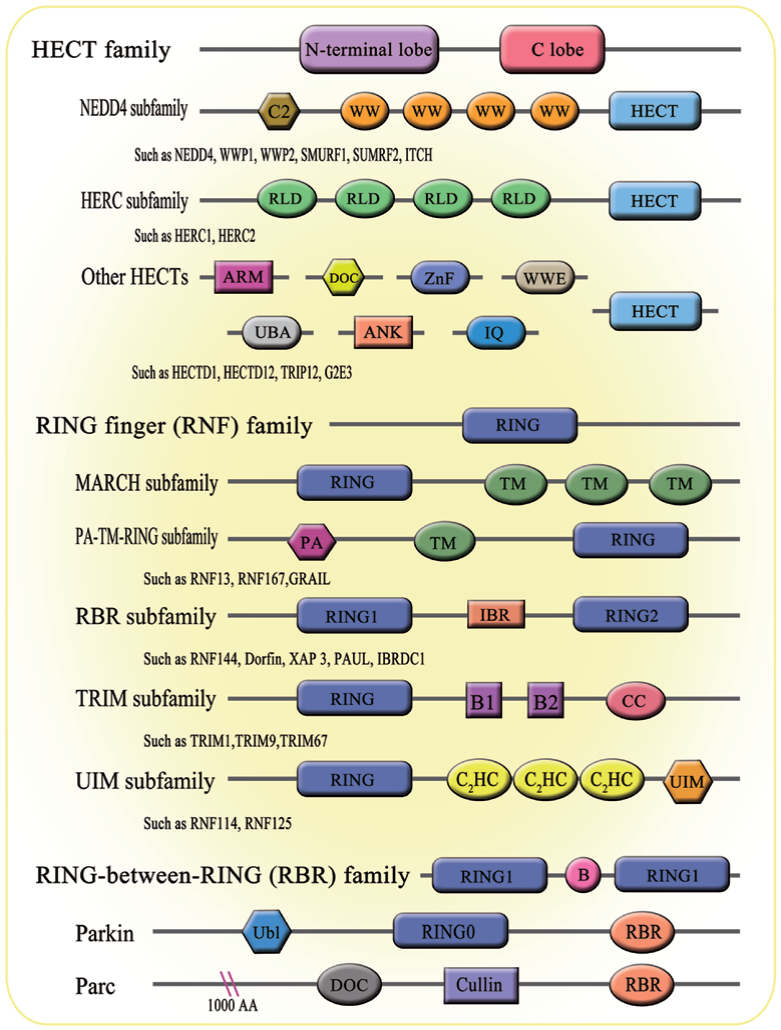
Classification of the Ub ligase E3. The HECT ligase can be divided into three subgroups based on the existence of the different domains it contains: i) NEDD4 family, including the C2 domain, 2-4 WW domain and HECT; ii) HERC family, including HECT and RCC1-like domains, E3 at least one or more RLDs; and iii) other families containing variable number and types of domains. All members of the RNF family are characterized by the N-terminal RING domain. Each subfamily has its unique domains besides the conserved RING domain. The MARCH subfamily is characterized by the zero to more C-terminal TM domain. The PA-TM-RING subfamily is characterized by the PA domain, two TM domains and longer C-end tail extension. The RBR subfamily is characterized by the IBR domain. The TRIM subfamily is characterized by two BB domains and a CC domain. The UIM subfamily is characterized by a C2HC-type zinc finger, two C2H2-type zinc fingers and UIM. The name RBR derives from the existence of two RING domains, RING1 and RING2, which are separated by the IBR. Each member of the RBR E3 ligase contains other specific domains, such as Parkin, which also contains RING0 and Ub domains. Ub, ubiquitin; HECT, homology to E6AP C-Terminus; NEDD4, neural precursor cell expressed developmentally downregulated protein 4; RLD, RCC1-like domain; RING, really interesting new gene figure; MARCH, Membrane-associated RING-CH; PA, protease-associated; TM, transmembrane; RBR, RING-between-RING; IBR, in-between-RING domain; UIM, ubiquitin-interacting motifs.

**Figure 3:**
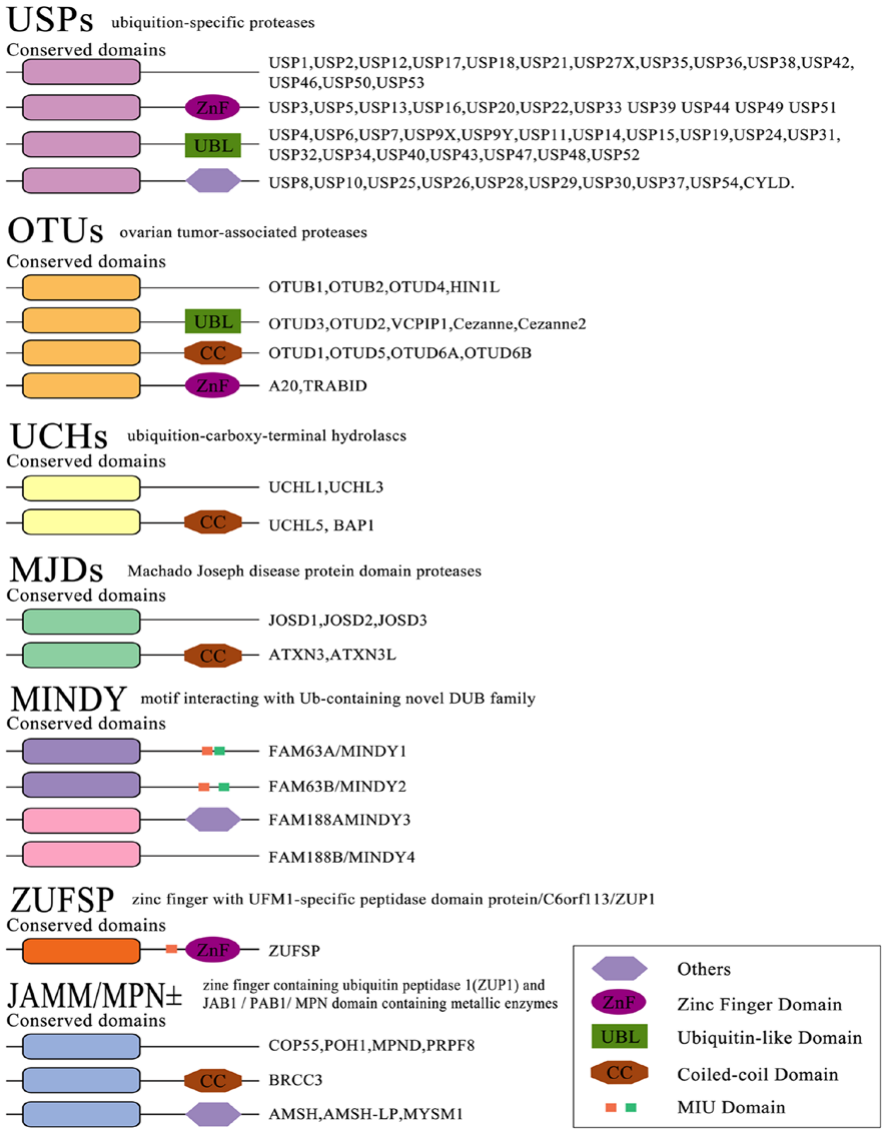
Classification of the DeUb enzyme family. The human genome encodes ~100 deubiquitinases, which are divided into six families: i) The USP family; ii) the UCH family; iii) the OUT; the MJD family; iv) the MINDY family; v) the ZUP1 and vi) the JAMM/MPN family. Ub, ubiquitin; USP, ubiquitin-specific protease; UCH, ubiquitin carboxy-terminal hydrolase; OTU, ovarian tumor-related protease; MJD, Machado Joseph disease protein domain protease; MINDY, motif interacting with ubiquitin-containing novel DUB family; DUB, deubiquitinating enzyme; ZUP1, zinc finger containing ubiquitin peptidase; JAMM/MPN, JAB1/PAB1/MPN domain containing metallic enzymes.

Ubiquitination is a conserved, widespread and dynamic post-translational modification involved in regulating the activity and stability of thousands of proteins, thereby affecting most cellular functions and responses (14). Ubiquitylation is a process mediated by the covalent attachment of ubiquitin (Ub) to the target protein, catalyzed by a series of enzymatic cascades, including E1-mediating activation, E2-mediating coupling and E3-mediating connection step (Fig. 1) (15). E3 then targets the protein by promoting the formation of an isopeptide bond between the C-terminal carboxyl group of Ub and one of the seven lysine residues of Ub (K6/11/27/29/33/48/63) or the N-terminal methionine of Ub (M1) (16-18). Subsequently, modified substrate proteins are degraded by 26S protein complexes or exhibit cellular function (19,20). E3 provides the reaction rate and substrate specificity for this cascade reaction. Accumulating evidence has shown that E3 ligases are associated with a number of biological processes, including bone homeostasis (21). There are 600-1,000 E3 ligases encoded by the human genome. According to the domain structure and ubiquitination mechanism through which E3 ligases interact with target proteins, E3 members are broadly divided into three major subfamilies: i) Homology to E6AP C-Terminus (HECT) family; ii) really interesting new gene figure (RING) family; and iii) RING-between-RING (RBR; Fig. 2). The reversibility of ubiquitination modification is achieved by deubiquitinating enzymes (DUBs) (22). At present, >100 DUBs are encoded in the human genome based on the catalytic region. They can be classified into two categories or six subfamilies (Fig. 3) (23-28).

**Figure 4:**
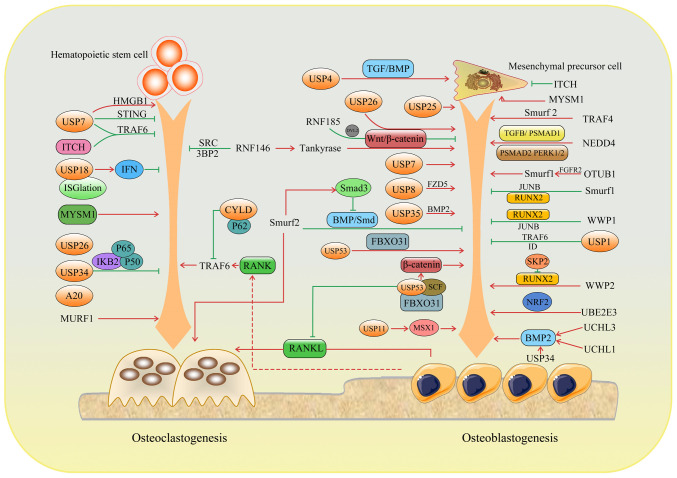
Mechanisms and effects of E3 ligases and DUBs on bone remodeling. In the process of differentiation of mesenchymal stem cells into osteoblasts and hematopoietic stem cells into osteoclasts, E3 enzymes and deubiquitinases regulate bone homeostasis through various mechanisms. DUBs, deubiquitinating enzymes; USP, Ub-specific proteases; ITCH, itchy E3 ubiquitin protein ligase; MYSM1, MPN domain containing 1; TRAF, tumor necrosis factor receptor-associated factor; HMGB1, high mobility group box 1; STING, stimulator of interferon genes; NEDD4, neural precursor cell expressed developmentally downregulated protein 4; RUNX, runt-related transcription factor; WWP, WW domain-containing E3 Ub protein ligase; OTUB1, ovarian tumor domain-containing ubiquitin aldehyde-binding protein 1; UCHL, Ubiquitin C-terminal hydrolase L; NRF2, nuclear factor (erythroid derived 2)-like 2.

In recent years, a growing body of evidence suggests that ubiquitination plays an important regulatory role in the maintenance of bone homeostasis. Osteoporosis and other related bone diseases are accompanied by abnormal expression and dysfunction of ubiquitination and de-ubiquitination enzymes. As a potential research hotspot for bone metabolism-related diseases and a new target for drug development, ubiquitination has a broad prospect in the field of osteoporosis prevention and treatment. The current review discusses the specific mechanism and role of ubiquitination in bone remodeling to provide a more theoretical basis and practical guidance for the treatment of related diseases (Fig. 4; Table I).

## 2. E3 Ub ligases and the bone

In a growing number of studies, bone remodeling has been linked to ubiquitination. E3 Ub ligases are mainly involved in osteoblast differentiation and bone formation. In the present review, the function and regulation of E3 ligases in bone homeostasis is discussed.

### Smad ubiquitination regulatory factor (Smurf) 1

Belonging to the C2-WW-HECT region Ub ligases, Smurf1 downregulates the level of alkaline phosphatase and osteocalcin and suppresses the osteogenic activity of osteoblasts, resulting in osteoporosis and fragility fractures. Al-Rawi *et al* (29) found that the micro duplication of the Smurf1 gene led to the first case of childhood osteoporosis. Mechanically, Smurf1 affects the process of osteogenic differentiation through various mechanisms. Runt-related transcription factor 2 (Runx2) is a core transcription factor for directed differentiation of the osteoblast cell lineage and plays a crucial role in promoting osteoblast differentiation. It is involved in the Ub-proteasome pathway, which regulates osteoblast differentiation and bone formation by interacting with and ubiquitinating Runx2 (30). The BMP signaling pathway plays a critical biological role in bone development and post-natal bone formation, mainly by regulating the differentiation and function of mesenchymal stem cells (MSCs) and osteoblasts. Smurf1 degrades BMP downstream signaling molecules also in an Ub-dependent manner and when Smurf1 is inhibited, bone formation is enhanced in osteoporotic mice with markedly age-related osteoporosis (31). Jun B proto-oncogene (JunB) is an ubiquitinated substrate of Smurf1 and a stimulator of MSC proliferation and differentiation into osteoblasts. Smurf1 is able to bind to and ubiquitinate JunB, which is subsequently degraded by the proteasome. This degradation process reduces the protein level of JunB, which inhibits its transcriptional activity and further affects the proliferation and differentiation of MSCs, leading to the regulation of bone metabolism (32). Additionally, the P53/microRNA (miR/miRNA)-17/Smurf1 pathway inhibits osteogenesis through suppressing MSC function in age-related models, leading to osteoporosis (33). However, the underlying mechanism remains elusive.

### Smurf2

Smurf1 and 2 share ~70% sequence homology and similar structural features (34), but Smurf2 has an additional WW structural domain, meaning that the functionality is different. Compared with Smurf1^−/−^ mice, the osteoclast number in Smurf2^−/−^ mice notably increased resulting in severe osteoporosis (35). Transforming growth factor-β (TGF-β) signaling is involved in the maintenance of bone homeostasis; TGF-β induces osteoblast differentiation and proliferation, inhibiting osteoclast formation (36). Smurf2 is transmitted primarily by TGF-β signaling, which is an important inhibitor of TGF-β. Smurf2 ubiquitinates the TGF-β receptor and Smad proteins to regulate the TGF-β signaling pathway and maintain tissue homeostasis (37). Furthermore, BMSCs from Smurf2^−/−^ mice showed increased osteogenesis, enhancing the BMP/Smad signaling pathway. More specifically, Smad3 ubiquitination by Smurf2 negatively regulates BMP/Smad signaling (38). In the skeletal system, Smurf2 has been investigated and it was shown to play an important regulatory role in the transfer of information between osteoblasts and osteoclasts. More specifically, Smurf2 is able to regulate the activity of osteoclasts, which are dependent on osteoblasts. This means that Smurf2 does not act directly on osteoclasts, but indirectly regulates osteoclast behavior by affecting osteoblasts. This regulatory mechanism may involve signaling and interactions between osteoblasts and osteoclasts to maintain homeostasis in the skeletal system (35).

### WW domain-containing E3 Ub protein ligase 1 (WWP1)

WWP1 is a 922-amino acid long HECT type E3 Ub protein ligase that interacts with the proline-rich polypeptide motif of target proteins to regulate protein transport, degradation, signaling and transcription. The role of WWP1 in bone homeostasis was first discovered in 2006 by Shu *et al* (39). WWP1 can polyubiquitinate Runx2 and decrease the differentiation and migration of osteoblasts. The role of the intracellular adaptor protein Schnurri-3 (Shn3) in osteoblastic bone formation is well established. Shn3 is able to regulate Runx2 protein levels through a delicate mechanism. Specifically, Shn-3 is able to interact with WWP1 and recruit it to Runx2. Once WWP1 binds to Runx2, it is able to promote the ubiquitination of Runx2, which in turn triggers the process of Runx2 degradation. In this way, Shn3 effectively controls the intracellular levels of the Runx2 protein, indirectly affecting the process of bone tissue formation and mineralization (40). Additionally, during inflammation-mediated osteoporosis models, WWP1 targets JunB for ubiquitination and degradation in MSCs to inhibit MSC differentiation into osteoblasts (41). As with WWP1, WWP2 belongs to the HECT type of E3 Ub protein ligases. WWP2 mediates monoubiquitination of Runx2 leading to its transactivation and resulting in increased osteogenic activity and promotion of osteogenic differentiation (42).

### RNF146

The RING finger (RNF) 146, a RING domain and PAR-acylation dependent cytoplasmic E3 ligase, is indispensable in bone formation. RNF146 can improve MSC self-renewal and osteogenesis/adipogenesis, leading to increased bone mass (43). In osteoclast differentiation, RNF146 can promote the ubiquitination and degradation of AXIN, thereby regulating the Wnt signaling pathway and activating β-catenin to promote osteoclast differentiation. In addition, BP2 activity is indispensable for SRC tyrosine kinase in osteoclast differentiation. RNF146 deubiquitinates 3BP2, thereby inhibiting SRC activity and reducing osteoclast differentiation ability. Furthermore, RNF146 expression can be suppressed by RANKL through the NF-κB pathway, by regulating the stability of AXIN and 3BP2, thereby intervening in osteoclast differentiation (44).

### RNF185

RNF185, also known as FLJ38628, is a RING domain E3 Ub ligase. In a high-throughput screening (45) of synthetic silencing RNA libraries targeting 5,000 human genes, RNF185 was identified as an endogenous inhibitor of osteoblastic norms. Deficiency of RNF185 can initiate human MSCs to differentiate into osteoblasts. An additional study further revealed the underlying mechanism through which RNF185 negatively regulates osteogenesis by degrading dishevelled (Dvl) 2 and downregulating the Wnt signaling pathway (46).

### Itchy E3 ubiquitin protein ligase (ITCH)

In bone metabolism, ITCH negatively regulates the differentiation of mesenchymal progenitor cells into osteoblasts by mediating the proteasomal degradation of the JunB protein, a positive osteoblast regulator (47). In addition, in animal experiments, high expression of ITCH was detected in mouse fracture callus. Several positive regulatory factors for osteoblast differentiation, including Runx2, increase in the callus of Itch^−/−^ mice fractures and thus the differentiation of osteoblasts is also markedly enhanced (48). In osteoclast formation, ITCH inhibits TRAF6 de-ubiquitination by binding the deubiquitinating enzyme cylindromatosis to inhibit RANKL-induced osteoclast. ITCH transcription is directly regulated by RANKL during osteoclast differentiation via the NF-κB (49).

### Others

S-phase kinase-associated protein (SKP) 2 regulates a wide range of cell cycle proteins by targeting the SKP1-Cullin1-F-box for ubiquitination and proteasomal degradation (50). SKP2 negatively targets Runx2 for Ub-mediated degradation to inhibit osteogenic differentiation (51). Muscle-specific RING finger E3 ligase 1 (MURF1), a RING-type E3 Ub ligase, exhibits a critical role in inducing skeletal muscle atrophy. It has been reported that prolonged bed rest for various reasons, such as fractures, spinal cord injury, brain disease and exposure to microgravity, causes loss of muscle mass and strength, which further leads to bone loss. Mechanistically, MURF1 is upregulated and increases protein degradation and muscle wasting in numerous muscle atrophy models (52). The suppression of MURF1 can reduce osteoclastogenesis (53). Ubiquitin-conjugating enzyme E2 E3 (UBE2E3) is negatively and positively associated with age and osteogenic genes, respectively. The lack of UBE2E3 was further shown to promote osteoporosis development and overexpression could inhibit cell aging and increase osteogenic differentiation of old BMSCs (54). TRAF4 is markedly reduced in bone sections of ovariectomized rats with local osteoporosis and patients with osteoporosis (55). Further mechanisms indicate that the TRAF4 degrades Smurf2 to enhance osteoblastic differentiation of MSCs. Neural precursor cell expressed developmentally downregulated protein 4 (NEDD4), a member of the HECT domain family, participates in cell proliferation, apoptosis, autophagy and other biological processes to regulate mammalian growth and development (56,57). NEDD4 enhances osteoblast proliferation and bone mass accumulation by inducing the degradation of TGF-1β-activated pSMAD1 and improving the activation of the pSMAD2 and pERK1/2 pathways (58).

## 3. Role of DUBs in bone remodeling

DUBs are involved in DNA repair, gene transcription, apoptosis, autophagy, DNA repair and immune responses (19). DUBs act as critical regulators for bone remodeling by regulating basic multicellular unit differentiation and/or function.

### Ub-specific proteases (USPs) and osteoblasts

Human adipose-derived stem cells (hASCs) can be extracted and isolated from human adipose tissue and have the potential for self-renewal and multidirectional differentiation. hASCs can be induced to differentiate in a variety of directions, such as adipogenic, osteogenic and chondrogenic. During osteogenic differentiation of hASCs, the expression level of USP7 changes and is positively associated with the degree of osteogenic differentiation. This implies that USP7 may play an important role in the osteogenic differentiation of hASCs (59). USP1 controls the NF-κB signaling pathway by inhibiting TRAF6 ubiquitination, thereby inhibiting osteoblast pyroptosis (60). USP1 deletion results in osteogenic impairment in mice and marked osteopenia. Overexpression of USP1 in MSCs inhibits osteogenic differentiation and promotes the preservation of MSC features. The mechanism is that USP1 deubiquitinated inhibitors of DNA-binding proteins to promote the preservation of MSC signatures (61). USP8 in bone progenitor cells prevents the degradation of the Frizzy-5 ubiquitinated Wnt receptor to ensure Wnt-induced osteogenesis, thus promoting bone formation (62). USP11 can stabilize the Msh homeobox 1 (MSX1) protein to upregulate the expression of osteogenic differentiation-related markers in human MSCs, consequently enhancing osteogenic differentiation (63). The USP34 protein is involved in bone formation by modulating BMP2 signaling in MSCs. When BMSCs or pre-osteoblasts are conditionally knocked out of USP34, mice have low bone mass. Mechanically, USP34 stabilizes both Smad1 and RUNX2 for osteogenic differentiation and bone formation (64). Ubiquitin C-terminal hydrolase-L3 (UCHL3) regulates BMP2-induced Smad1 polyubiquitination to promote osteoblast differentiation (65).

### USP4

USP4 affects osteogenic differentiation through multiple mechanisms. The ubiquitination of the Dvl protein can activate the Wnt/β-catenin signaling pathway to affect osteoblast differentiation (66). By removing the lysine-63-linked polyUb chain from Dvl, USP4 strongly inhibits Wnt/β-catenin signal transduction to weaken osteogenic differentiation (67). Regulatory pathways associated with TGF/BMP are also important in bone growth (68). USP4 can target the TGF-β receptor for deubiquitination and enhance the activation of the TGF-β/BMP signaling pathway to promote the proliferation and differentiation of MSCs (69).

### USP53

USP53 enhances osteogenic function and differentiation in human BMSCs by stabilizing key proteins associated with osteogenic differentiation and regulating the BMP signaling pathway. Moreover, USP53-overexpressing human BMSCs markedly promote bone regeneration in murine calvaria bone defects. Mechanistically, the interaction of USP53 with F-box protein 31 revealed a new role for USP53 in cellular signaling and protein degradation. This interaction specifically involves the degradation of β-catenin by the Skp1/Cul1/F-box protein complex, a key process that is essential for the regulation of the Wnt signaling pathway (70). In addition, USP53 acting on the vitamin D-receptor-SMAD3 pathway increases RANKL-dependent osteoclastogenesis through osteoblasts (71).

### UCHL1

UCHL1 was originally identified in neurons. CXCL7, a chemokine with a C-X-C motif, is associated with the posterior longitudinal ligament ossification of the spine (OPLL) phenotype, manifested by dyskinesia, ectopic ossification of posterior ligament tissue and osteoporosis. UCHL1 is overexpressed in the serum of mice with a CXCL7 gene deletion and in patients with OPLL (72). In patients with periodontitis, UCHL1 leads to periodontal ligament stem cell osteogenesis and alveolar bone loss by regulating the BMP2/Smad signaling pathway. However, it is unclear which molecule is deubiquitinated by UCHL1. By inhibiting the high expression of UCHL1, periodontal bone loss is reduced and inflammation is alleviated (73).

### USPs and osteoclasts

#### Cylindromatosis (CYLD)

CYLD negatively regulates osteoclastogenesis. Consistently, the number and activity of osteoclasts in CYLD knockout mice increases and the mice exhibit severe osteoporosis (74). With the use of proteasome inhibitors, CYLD accumulation increases, leading to impairment of osteoclast production and function (75). Mechanically, CYLD, through deubiquitination, is able to remove the Ub chain on TRAF6 and inactivate it, thereby inhibiting the activation of the RANK signaling pathway (76).

#### A20

A20 has been shown to mainly regulate osteoclast formation. A20 overexpression has anti-inflammatory effects on human periodontal ligament cells (HPDLCs) and blocks osteoclast differentiation (77). A20 knockout mice have increased osteoclasts, thinner bone trabeculae and are prone to develop severe osteoporosis (78). A20 induction by intravenous gamma globulin attenuates RANKL inducing NF-κB signaling to reduce osteoclastogenesis *in vivo* as well as inhibits bone resorption, thus preserving bone mass (79). Mechanistically, the A20 anti-osteoclast function is mainly dependent on inhibiting NF-κB activation by altering IκBα ubiquitination-mediated degradation, which physically interacts with NF-κB to inhibit its activation (80). TRAF6-dependent autophagy in HPDLCs is inhibited for the reduced expression of A20 with 2% hypoxia treatment, resulting in decreased osteoclast differentiation and formation. The underlying mechanism involves A20 inhibiting K63-linked, while it enhances K48-based ubiquitination of TRAF6 to suppress TRAF6 activation and promote TRAF6 proteasomal degradation, respectively, thus inhibiting autophagy under hypoxic conditions (81).

#### Jab1/Mpr1/Pad1 N-termina domain associated metalloisopeptidase (JAMM/MPN)

Myb-like, Swi3p, Rsc8p and Moira (SWIRM) and MPN domain containing 1 (MYSM1) is a metalloprotease composed of three domains, MPN, SWI2/SNF2 ISWI-like SANT (SANT) and SWIRM (82). MYSM1 is not only an important regulator of hematopoiesis and immunity (83), but also essential for maintenance of bone homeostasis. Mysm1^−/−^ mice showed enhanced autonomic differentiation of MSCs and accelerated adipogenesis, as evidenced by reduced bone mass in long bones and cranial bones (84). A study demonstrated that osteoclast progenitor cells in Mysm1^−/−^ mice showed decreased proliferative ability and markedly decreased osteoclast number and absorption activity (85). However, the underlying mechanism of MYSM1 in osteoclast differentiation and function remains enigmatic.

#### Others

USP7 has a dual effect on osteoclasts. On the one hand, in CD14^+^ human peripheral blood mononuclear cell studies, USP7 promotes osteoclast differentiation through high mobility group box 1 (HMGB1) deubiquitination (86). On the other hand, USP7 negatively regulates osteoclastogenesis through a mixed mechanism of deubiquitination-mediated TRAF6 signaling and deubiquitination-mediated degradation of stimulator of interferon genes (STING) signaling (87). USP18 specifically removes the ISGylation from substrate proteins by interferon-stimulated (ISG) gene 15 and also regulates the interferon signaling pathway to block ISGs (88,89). The differentiation ability and bone resorption activity are increased in USP18-deficient mice, resulting in an osteoporotic phenotype in mice (90). USP34 is not only required for osteogenic differentiation, but also inhibits osteoclastogenesis. USP34 deficiency leads to RANKL-mediated NF-κB activation and increased osteoclast production, which in turn leads to a phenotype of bone loss in mice. The specific process of this mechanism is as follows: Under normal conditions, USP34 inhibits the activation of the NF-κB pathway by stabilizing IκBα, a protein that inhibits NF-κB, through deubiquitination. However, in the absence of USP34, the stability of IκBα is reduced, resulting in excessive activation of the NF-κB pathway. This activated state enables RANKL to bind easily to its receptor RANK, which in turn promotes osteoclast generation and bone resorption function (91). USP26 plays a balancing role during osteogenic and osteoclast differentiation. USP26 enhances IκBα stability to inhibit NF-κB activation, consequently suppressing osteoclast differentiation in bone marrow-derived macrophages (92). Moreover, in bone defect mice, USP26 increases osteogenic differentiation, promotes bone regeneration and reduces bone loss (92). OTU domain-containing Ub aldehyde-binding protein 1 in osteoblasts antagonizes the ubiquitination of Fibroblast Growth Factor Receptor 2 (FGFR2) mediated by the Ub ligase SMURF1 in an enzyme activity-independent manner to maintain protein stability, resulting in enhanced osteoblast formation (93).

## 4. Medication for osteoporosis

The main goal of osteoporosis medications is to reduce the risk of osteoporotic fractures and improve the quality of life of patients by relieving pain, increasing bone density and preventing fractures (94). According to the different mechanisms of action, anti-osteoporosis drugs are generally placed in three categories: i) Inhibitors of bone resorption; ii) promoters of bone formation; and iii) dual-acting drugs. Bone formation promoters are mainly parathyroid hormone receptor agonists, including teriparatide and appalatide. Sclerostatin monoclonal is a double-acting drug (95). There are five main categories of bone resorption inhibitors: i) Bisphosphonates (BPs); ii) RANKL inhibitors; iii) estrogen; iv) selective estrogen receptor modulators; and iv) calcitonin (Table II).

BPs are widely used in the treatment of osteoporosis, where they are effective in inhibiting bone resorption and increasing bone mass at vertebral, non-vertebral and hip sites, thereby reducing the risk of fracture (96). However, oral BPs can increase upper gastrointestinal symptoms in patients and ~33% of patients receiving first intravenous zoledronic acid develop flu-like symptoms. In addition, prolonged use of BP medications may result in reduced blood supply to the jaws, thereby increasing the risk of osteonecrosis of the jaws as well as inhibition of bone turnover, leading to impaired bone remodeling, thereby increasing the risk of atypical fractures (97). Denosumab is also the most common osteoporosis drug in the clinic. It can be combined with RANKL to markedly reduce the degree of osteoporosis in patients. If Denosumab was stopped without other osteoporosis drugs, bone mineral density showed a steep decline (98). It is noteworthy that rashes, cellulitis, femoral shaft or subtrochanteric fractures and jaw necrosis may occur during use. The application of estrogen in women aged <60 years or 10 years after menopause can effectively prevent bone loss caused by menopause. Estrogen acts by indirectly regulating calcitonin, parathyroid hormone and 1,25-(OH)2D3. However, Estrogen replacement therapy has been limited clinically due to potential adverse effects, including venous thromboembolism, cerebrovascular disease, stroke and breast cancer (99). Menopausal hormone therapy (MHT) has its unique advantages in the treatment of postmenopausal osteoporosis (100,101). In the mid-1990s and early 2000s, in most European and North American countries, MHT is widely used to prevent the risk of postmenopausal osteoporosis and related bone fractures in women. Following the publication of the randomized Women's Health Initiative (WHI) report (102), which found that MHT increased the risk of coronary heart disease, stroke and breast cancer, there was a dramatic decline in MHT prescribing worldwide. The current trend is to recommend lower doses of estrogen for effective relief of vasomotor symptoms and prevent vaginal atrophy with an improved bleeding profile than with higher estrogen dosages within 10 years of menopause than was recommended a few years ago. For the prevention of endometrial cancer caused by MHT, it is recommended to add progesterone (103). In addition, compared with oral estrogen therapy, transdermal estrogen patch can reduce the risk of venous thrombosis and atherosclerotic vascular disease and also protect the heart (104). Therefore, low-dose non-oral MHT may be considered for women with cardiovascular risk factors, older women and those who choose to use MHT for extended periods to protect bone (105,106). In conclusion, individualized treatment is advocated for hormone replacement therapy and different formulations, dosages and MHT regimens are considered at the same time. The artificial synthesis of selective estrogen receptor modulators can selectively act on different tissue estrogen receptors to reduce the incidence of breast cancer in women (107). However, some side effects, including vasomotor symptoms, muscle spasm and venous thrombosis, remain. The Use of Raloxifene in Heart Disease (RUTH) study showed that Raloxifene increased fatal stroke (0.7‰ increase in absolute risk) and the risk of the occurrence of venous thromboembolic disease (absolute risk increase by 1.3‰) (108).

Parathyroid hormone is one of the important hormones that regulate calcium and phosphorus metabolism, as well as bone conversion. It has been shown that parathyroid hormone promotes bone anabolic metabolism that is dependent on low-dose and intermittent administration. The duration and dosage of teriparatide use are closely associated with rat bone tumors (109), but post-marketing surveillance did not reveal a higher than expected risk of osteosarcoma (110). Calcitonin reduces blood calcium and phosphorus mainly by inhibiting osteoclasts. However, long-term use of calcitonin leads to clinical resistance. In addition, there may be an increased risk of cardiovascular complications. This may be associated with the effect of calcitonin on the cardiovascular system; it may affect the systolic and diastolic function of the heart, or the relaxation and contraction of blood vessels (111). Monoclonal sclerostin romosozumab promotes the formation of bone mass, while it inhibits the resorption of bone, rapidly forming bone on trabecular and cortical bone surfaces to increase bone density and strength (112). The most common adverse reactions include arthralgia, nasopharyngitis, back pain and injection site reactions, rare mandibular osteonecrosis and atypical fractures. The black box warning on the potential cardiovascular risks of romosozumab, including serious adverse events such as non-fatal myocardial infarction, non-fatal stroke and cardiovascular death, may indeed place some limitations on its clinical use (95). Osteoporosis guidelines emphasize vitamin D and calcium supplementation as basic measures. Importantly, it has been shown that when patients with osteoporosis are treated with anti-osteoporosis drugs, the effectiveness of these drugs is markedly reduced without adequate vitamin D and calcium supplementation (113).

## 5. Clinical application and prospects of UPS

UPS is critical for bone cell formation and differentiation. Based on these mechanisms of action, drugs targeting these Ub ligases and deubiquitinating enzymes for the treatment of osteoporosis and other related bone diseases continue to be developed. For example, by inhibiting the activity of certain Ub ligases, it may be possible to promote bone formation or inhibit bone resorption for the treatment of osteoporosis. By contrast, by activating the activity of certain deubiquitinating enzymes, it may also be possible to have a positive impact on the treatment of skeletal diseases.

Catalpol, a biologically active cyclic enol ether terpene glucoside extracted from the traditional Chinese Medicine *Rehmannia glutinosa*, inhibits the ubiquitination and degradation of phosphatase and tensin homolog to increase phosphatase activity and then inhibits downstream AKT and NF-κB associated with RANKL signal pathways, consequently suppressing osteoclast formation and bone absorption (114). In mice with LPS and ovariectomized-induced bone loss, catalpol improves bone loss by attenuating osteoclast activity (114). Melatonin is a biologically active amine hormone secreted by the pineal gland of the brain and is a signaling molecule with a circadian rhythm, participating in various physiological processes including bone metabolism (109). Melatonin inhibits RANKL receptor activators to inhibit osteolysis and prevent bone loss and also promotes osteoblast differentiation and activity by melatonin receptor 2 (115). Melatonin reduces the ubiquitination of the Smad1 protein preventing its degradation by inhibiting Smurf1 activity, thereby stabilizing BMP Smad1 signaling activity and restoring inflammatory factor-induced osteogenesis (116). Carnosic acid (CA), an amino acid substance with strong antioxidant and anti-aging effects, is widely used in weight loss foods, nutritional supplements and other fields. CA binds to the ligand-binding region of cholesterol and estrogen receptor-associated receptor α, promoting its ubiquitination and proteasomal degradation, thereby inhibiting RANKL-associated osteoclast formation and ovariectomy-induced bone loss (117).

Beraprost can improve postmenopausal osteoporosis by downregulating the NEDD4-mediated ubiquitination-proteasome degradation of Runx2 (118). The selective complexes B06 and B75 block binding of Smurf1 to Ub, thereby stabilizing the level of the BMP signaling component Smad1/5 protein and enhancing osteoblast activity. This also offers promising new clinical prospects for the therapy of osteoporosis (119). miR-195-5p can target and inhibit the activation of BMP-2/SMAD/Akt/Runx2 pathway signaling by Smurf1, to promote osteogenic differentiation in ovariectomized mouse models (120). Chalcone derivatives enhance local bone mass after lumbar spine fusion surgery in normal BMP-2 mice with high expression of Smurf1 (31). Bortezomib, is a 26S proteasome inhibitor which inhibits the degradation of polyUb protein by suppressing the hydrolysis activity of the proteasome. Bortezomib regulates proteasomal degradation of Runx2 via the Wnt/β-catenin signaling pathway (121), inducing osteogenic differentiation and promoting osteogenesis (122). Furthermore, a recent study suggested that bortezomib may treat post-menopausal osteoporosis by inhibiting the Smurf-mediated ubiquitination-proteasome process (123). Withaferin A, also a proteasome inhibitor, promotes osteoblast proliferation and differentiation as well as regulates bone anabolism to promote osteoporotic fracture healing (124).

miRNAs participate in the progress in osteoporosis through a complex signaling pathway network (125). miR-21-5p inhibits osteoclast differentiation by targeting SKP2. In an ovariectomy (OVX) mouse model injected with miR-21-5p, the number of osteoclasts was reduced and the degree of osteoporosis was improved (126). Neat1, the mechanoreceptor long non-coding RNA (lncRNA), under mechanical stimulation, regulates osteogenic function by nuclear retention of the paraplaque-dependent E3 Ub ligase Smurf1 mRNA. In Neat1-knockout hindlimb unloading mice, no bone damage or bone loss are found. This provides a treatment reference for bone loss caused by long-term space flight or inactivity and osteoporosis associated with aging (127). In addition, SNHG1 lncRNA inhibits osteogenic differentiation through NEDD4-mediated ubiquitination by suppressing the activation of the p38-MAPK signaling pathway, a key trigger factor in bone formation (128). In recent years, a new drug delivery system, nanoparticles, has been used to treat osteoporosis. Ferumoxytol and ferucarbotran, as clinical nanoparticles, exert anti-osteoporosis effects by inhibiting osteoclastogenesis and participating in bone metabolism. Nanoparticles trigger the upregulation of p62, resulting in the recruitment of CYLD to enhance deubiquitination and inactivation of TRAF6, the main controller of the RANKL and NF-κB signal (129). When downstream activation of the NF-κB signaling pathway and the MAPK signaling pathway is attenuated, the proliferation and differentiation processes of osteoclast precursors may be impeded, leading to a reduction in osteoclast formation. Intravenous injection can improve bone quality in OVX mice and it is anticipated to be an alternative drug for the treatment of osteoporosis (129).

In addition, several clinically used drugs have been discovered to be involved in bone homeostasis via the UPS system, such as thalidomide (130), lansoprazole (131), vitisin A, clomimidazole and zoledronic acid (132), all of which inhibit or induce ubiquitination of related substrates.

## 6. Conclusion and future perspectives

The present review introduced and summarized the current literature on ubiquitination and deubiquitination in osteoporosis and bone homeostasis, highlighting the critical importance of Ub-dependent proteolytic systems in skeletal cell function. The E3 connecting enzymes are mainly regulated by transcription factors and they have notable protein participation in bone formation in the signaling pathway of BMSC and osteoblast proliferation and differentiation. Only a few studies have shown that RNF146 (44) and Smurf2 (35) regulate osteoclasts. The E3 protease system can influence the bone remodeling process by regulating different target proteins. They recognize and degrade proteins that are involved in bone formation, thereby regulating the rate and extent of bone formation. At the same time, they also recognize and degrade proteins involved in bone resorption, thereby controlling the process of bone resorption. This precise regulatory mechanism is essential for maintaining bone homeostasis.

Research on the regulation of deubiquitinating enzymes on osteoblasts and osteoclasts is limited and the mechanism and molecules of action have not been fully elucidated. To date, the relationship between MJDs and MINDY families with skeletal cell differentiation and function has not been reported. Elucidating the intrinsic mechanisms by which DUB gene alterations regulate osteoblast and osteoclast functions is important for the development of novel and more effective therapeutic strategies for osteoporosis. Designing drugs targeting specific genetic alterations in the DUB gene to avoid its dysregulation is one of the important directions for developing novel osteoporosis treatment strategies. Through in-depth study of the function and regulatory mechanisms of the DUB gene, potential drug targets can be identified and targeted drugs can be designed to regulate the function of osteoblasts and osteoclasts. These drugs can correct the imbalance of bone metabolism by altering the activity or expression level of the DUB gene, thus achieving the goal of treating bone diseases such as osteoporosis. In the future, through in-depth study of the function and regulatory mechanism of the DUB gene, as well as the design of drugs targeting its specific genetic alterations, it is anticipated to provide new ideas and methods for the treatment of skeletal diseases such as osteoporosis.

Meanwhile, several anti-osteoporosis drugs commonly used in the clinic were discussed in the present review as well as their disadvantages and the current clinical studies on ubiquitination-related drugs for osteoporosis. At present, anti-osteoporosis drugs have shown some efficacy in clinical research and their benefits outweigh the risk of side effects, but patients lack compliance due to the aforementioned side effects, high costs and sequential treatment. Although designing drugs to target specific genetic alterations in the DUB gene is challenging and the adverse effects and the interactions with other drugs are not yet clear, the future research direction may be to treat osteoporosis by mediating ubiquitination.

Furthermore, the internal mechanisms by which ubiquitination regulates skeletal cell function have not been thoroughly investigated and the specific sites of ubiquitination modification and the interactions of different molecules acting at the same site have not been clarified. Decoding the pathways between ubiquitination and other modification mechanisms has also not been described in depth. These practical mechanisms should be further investigated in the future.

## Figures and Tables

**Table I:** E3 ubiquitin ligases, deubiquitinases and bone remodeling.

Family	Enzyme	Mechanism	Function	(Refs.)
HECT family	Smurf1	Interacts with Runx2 in a ubiquitin proteasome dependent manner, mediating its degradation	Inhibits osteoblast differentiation and mineralization	(27)
		Degrades BMP downstream signaling molecules		(28)
		Control JunB turnover		(29)
		Involved in P53/miR-17/Smurf1 pathway		(30)
	Smurf2	Ubiquitinates TGF-β receptor and Smad proteins	Inhibits osteoblast differentiation and proliferation and enhances osteoclast formation	(33,34)
		Inhibits the BMP/Smad signaling pathway	Inhibits osteogenesis	(35)
		Information transmission between osteoblasts and osteoclast	Regulates the activity of osteoblast dependent osteoclast	(32)
	WWP1	Mediates Runx2 degradation		(36,37)
		Targets JunB for ubiquitination and degradation in MSCs	Inhibits osteogenic differentiation	(38)
	WWP2	Mediates mono-ubiquitination of Runx2	Enhances osteogenic differentiation	(39)
	ITCH	Mechanism unknown	Inhibits osteogenic differentiation	(45,46)
		Limits TRAF6 deubiquitination by binding the cylindromatosis	Inhibits osteoclastogenesis	(47)
	NEDD4	Induces degradation of TGF-1β-activated pSMAD1 and improves the activation of pSMAD2 and pERK1/2 pathways	Enhances osteoblast proliferation and bone mass accumulation	(54-56)
RING family	RNF146	Mediates the PARsylation of Tankyrase to stimulate Wnt pathway via degrading Axin.	Enhances osteogenic differentiation	(40,41)
		Regulates the stability of SRC and 3BP2, the adaptor protein essential for OCs	Inhibits osteoclastogenesis	(42)
	RNF185	Regulates Wnt signaling pathway by degrading dishevelled 2	Inhibit osteogenic differentiation	(43,44)
	SKP2	Negatively targets Runx2 for ubiquitin-mediated degradation	Inhibits osteogenic differentiation	(48,49)
	MURF1	Mechanism unknown	Promotes osteoclastogenesis	(50,51)
	TRAF4	Degrades Smurf2	Promotes osteogenic differentiation	(53)
Ubiquitin conjugating enzyme USP	UBE2E3	Might regulate the nuclear factor erythroid 2-related factor and control its function	Promotes osteogenic differentiation	(52)
	USP1	Acts on TRAF6 by suppressing TRAF6 ubiquitination to control NF-κB signaling	Inhibits osteoblastic pyroptosis	(61)
		Deubiquitinated ID proteins promote the preservation of mesenchymal stem cell signatures	Inhibits osteogenic differentiation	(62)
	USP4	Through Dishevelled inhibits Wnt/β-catenin signal transduction	Antagonizes osteoblast differentiation	(67,68)
		Targets TGF β receptor to enhance the activation of TGF/BMP signaling pathway	Promotes the proliferation and differentiation of MSCs	(69,70)
	USP7	Mechanism unknown	Promotes osteogenic differentiation of hASCs	(60)
		Binds to and deubiquitination of HMGB1	Promotes osteoclast differentiation	(87)
	USP8	Attenuates TRAF6/TAK1 Axis and stimulates STING Signaling	Inhibits osteoclastogenesis	(88)
		Prevents the degradation of the Frizzy-5 ubiquitinated Wnt receptor to ensure Wnt-induced osteogenesis	Promotes osteogenesis	(63)
	USP11	Stabilizes the MSX1 protein	Promotes the osteogenic differentiation	(64)
	USP18	Regulates Type I IFN signaling by deconjugating ISGlation	Inhibits osteoclastogenesis	(89-91)
	USP26	Stabilizes β-catenin to promote the osteogenic activity of MSCs	Promotes the osteogenic differentiation	(93)
		Enhances IκBα stability to inhibit NF-κB activation	Suppress osteoclast differentiation	
	USP34	Modulates BMP2 signaling in mesenchymal stem cells	Promotes osteogenic differentiation and bone formation	(66)
		Inhibits the NF-κB pathway by deubiquitinating and stabling IκBα	Inhibits osteoclastogenesis	(92)
	USP53	Interacts with F-box protein 31 to promote the degradation of β-catenin by Skp1/Cul1/F-box protein complex	Enhances the osteogenic and differentiation	(71)
		Act on vitamin D-receptor-SMAD3 pathway	Promotes osteogenesis	(72)
	CYLD	Negatively regulates the RANK signaling pathway and osteoclast formation by deubiquitinating and inactivating TRAF6	Inhibits osteoclastogenesis	(75-77)
UCHs	UCHL1	Regulates BMP2/Smad signal pathway	Promotes osteogenesis	(73,74)
	UCHL3	Regulates BMP2 induced Smad1 polyubiquitination	Promotes osteoblast differentiation	(66)
OTUs	A20	Attenuates RANKL inducing NF-κB signaling by altering IκB ubiquitination-mediated degradation	Inhibits osteoclast differentiation	(78-81)
		Via TRAF6-dependent autophagy in under hypoxia	Inhibits osteoclastogenesis	(82)
	OTUB1	Stabilizes FGFR2	Promotes osteogenesis	(94)
JAMM/MPN	MYSM1	Mechanism unknown	Promotes osteogenic differentiation	(85)
		Mechanism unknown	Increases osteoclast number and absorption activity	(86)

HECT, homology to E6AP C-Terminus; Runx, Runt-related transcription factor 2; BMP, bone morphogenetic protein; JunB, Jun B proto-oncogene; miR, microRNA; Smurf, Smad ubiquitination regulatory factor; WWP, WW domain-containing E3 Ub protein ligase; MSCs, mesenchymal cells; ITCH, itchy E3 ubiquitin protein ligase; TRAF, tumor necrosis factor receptor-associated factor; NEDD4, neural precursor cell expressed developmentally downregulated protein 4; RING, really interesting new gene figure; RNF, The RING finger; OCs, osteoclasts, SKP, S-phase kinase-associated protein; MURF, Muscle-specific RING finger E3 ligase; UBE2E3, Ubiquitin-conjugating enzyme E2 E3; USP, Ub-specific proteases; HMGB1, high mobility group box 1; TAK1, Transforming growth factor-β (TGF-β)-activated kinase 1; STING, stimulator of interferon genes; MSX1, Msh Homeobox 1; RANK, receptor activator of nuclear factor-κB; RANKL, RANK ligand; ovarian tumor domain-containing ubiquitin aldehyde-binding protein 1; FGFR2, Fibroblast Growth Factor Receptor; JAMM, Jab1/MPN domain associated metalloisopeptidase; MPN, Mpr1/Pad1 N-terminal; MYSM1, MPN domain containing 1.

**Table II:** Common anti osteoporosis medication guideline recommendations and precautions.

A, Bone resorption inhibitors					
Action mechanism	Drug type	Drug names	Guideline recommendation	Adverse reactions	Contraindications and important warnings
	Bisphosphonates	Alendronate, Isedronate, ibandronate, zoledronic acid	The first-line option and the most cost-effective interventions. Effectively reduce the risk of multiple fractures (vertebral, non-vertebral and hip)	Oral: Astrointestinal symptoms such as abdominal pain, diarrhea, nausea, dyspepsia and constipation; Intravenous: Influenza-like symptoms occur in 1/3 of patients within 3 days of medication	Zoledronic acid is prohibited (creatinine clearance below 35 ml/min). Preoperative improvement of oral problems may reduce the risk of osteonecrosis of the jaw. Oral for 5 years and intravenous for 3 years, re-evaluate and consider the drug holiday.
	RANK ligand inhibitor	Denosumab	Effectively reduces the risk of multiple fractures (vertebral, non-vertebral and hip) (96). Approved for use only in postmenopausal women in some countries.	Hycalcemia, severe infection (cystitis, respiratory tract infection, skin cellulitis, etc.), rash, skin pruritus, myalgia or bone pain, etc.; individual reports of jaw necrosis and atypical femur fracture.	Ensure that the patient knows the osteoporosis management plan. Desumamab cannot be easily stopped to avoid rapid decline of bone mineral density
	Estrogen	Estradiol, estropipate, conjugated oestrogen	Low-risk postmenopausal women	Headache, edema, tumor, thrombus	Increased risk of cardiovascular events and breast cancer. Initiation of treatment for more than 10 years after menopause is not recommended;
	Selective estrogen receptor modulators	Raloxifene bazedoxifene bazedoxifene and conjugated oestrogen	Reduces vertebral risk in postmenopausal osteoporotic women	Vasomotor symptoms, muscle spasm. Occasional venous thrombosis	Venous thromboembolism. Increased risk of stroke death in women at high risk of coronary heart disease. For women only.
	Calcitonin	(Human, salmon)	Osteoporosis and its induced	Face flushing, nausea, allergy bone pain	Note the allergy test
B, Bone formation promoters					
	Parathyroid hormone receptor agonist	Teriparatide and appalatide	Teriparatide is the first drug for postmenopausal osteoporosis, male and glucocorticoid-induced osteoporosis with high risk of fracture (97)	Nausea, limb pain, headache and vertigo	The duration of treatment should not exceed 24 months. Theoretically there is an increased risk of osteosarcoma (91). Contraindicated for patients with prior bone radiotherapy, bone malignancies, bone metastases or hypercalcemia
C, Dual-acting drugs					
	Sclerostin monoclonal antibody	Romosozumab	First-line treatment for women with high risk of postmenopausal fracture, especially for patients with spinal fracture	Arthralgia, nasopharyngitis, back pain and injection site reactions, rare mandibular osteonecrosis and atypical fractures	The duration of treatment should not exceed 24 months. Be aware of potential cardiovascular risks

RANK, receptor activator of nuclear factor-κB.

## Data Availability

Not applicable.
